# Impact of Selected Natural Bioactive Substances on Immune Response and Tight Junction Proteins in Broiler Chickens

**DOI:** 10.1002/vms3.70175

**Published:** 2025-02-28

**Authors:** Brigitta Csernus, Georgina Pesti‐Asbóth, Judit Remenyik, Sándor Biró, László Babinszky, László Stündl, János Oláh, Nóra Vass, Levente Czeglédi

**Affiliations:** ^1^ Department of Evolutionary Zoology and Human Biology University of Debrecen Debrecen Hungary; ^2^ Doctoral School of Animal Science University of Debrecen Debrecen Hungary; ^3^ Center for Complex Systems and Microbiome Innovations, Faculty of Agricultural and Food Sciences and Environmental Management University of Debrecen Debrecen Hungary; ^4^ Department of Human Genetics, Faculty of Medicine University of Debrecen Debrecen Hungary; ^5^ Department of Animal Nutrition Physiology, Institute of Animal Science, Biotechnology and Nature Conservation, Faculty of Agricultural and Food Sciences and Environmental Management University of Debrecen Debrecen Hungary; ^6^ Institute of Food Technology, Faculty of Agricultural and Food Sciences and Environmental Management University of Debrecen Debrecen Hungary; ^7^ Farm and Regional Research Institute of Debrecen University of Debrecen Debrecen Hungary; ^8^ Department of Animal Science, Institute of Animal Science, Biotechnology and Nature Conservation, Faculty of Agricultural and Food Sciences and Environmental Management University of Debrecen Debrecen Hungary

**Keywords:** broiler chicken, cytokine, immune response, immunoglobulin, natural bioactive compounds, tight junction proteins

## Abstract

This study was conducted to examine the effect of dietary natural compounds, such as β‐glucan, carotenoids, oligosaccharides and anthocyanins, on immune response and tight junction proteins in broiler chickens. A total of 900 one‐day‐old chickens were allocated to five treatments in three floor pens (replicates) of 60 broilers per pen. Chickens were fed five diets: a control (basal) diet, a diet supplemented with β‐glucan at 0.05%, or diets supplemented with carotenoids, oligosaccharides or anthocyanins at 0.5% of each compound. Male broilers were randomly selected for sample collections. On Day 25, plasma samples were collected from the brachial vein. On Day 26, six broilers were intraperitoneally injected with 2 mg of lipopolysaccharide per kg of body weight. Twelve hours later (Day 27), blood and ileum samples were collected to determine immune parameters and tight junction proteins using ELISA assays. The results showed that anthocyanin supplementation reduced the level of interleukin‐1β compared to the lipopolysaccharide‐injected control group (*p* = 0.047), which suggests that anthocyanin could partly alleviate the inflammation. Carotenoids reached a lower level of interleukin‐6 compared to the β‐glucan treatment (*p* = 0.0466). β‐Glucan (*p* = 0.0382) and oligosaccharides (*p* = 0.0449) increased the level of plasma immunoglobulin G compared to the challenged control group, which may indicate an enhanced humoral immunity. Furthermore, β‐glucan (except for occludin 2), carotenoids, oligosaccharides and anthocyanins increased (*p* < 0.05) the levels of ileal zonula occludens‐1, occludin 1 and occludin 2 compared to the lipopolysaccharide‐challenged control chickens. This may suggest that all the bioactive substances improved the gut barrier function. The plasma levels of tight junction proteins show higher concentrations in lipopolysaccharide‐challenged groups compared to the non‐challenged groups (*p* < 0.05). This may refer to the tight junction disruption and appearance in circulation as a reflection of lipopolysaccharide exposure.

## Graphical abstract

We defined that natural bioactive compounds, such as β‐glucan, carotenoids, oligosaccharides and anthocyanins, can be effective in mitigating inflammatory response and gut barrier impairment induced by lipopolysaccharide challenge in broiler chickens. Carotenoid and anthocyanin supplements can be suggested to alleviate inflammation, while β‐glucan and oligosaccharides can improve humoral immunity. Furthermore, all the studied natural compounds can enhance the levels of tight junction proteins, which suggests their beneficial effects on chickens’ gut health.

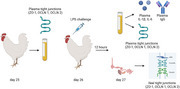

## 1 Introduction

Several stressors can affect the health status of broiler chickens, including immunological challenges or oxidative stress, which results in impairments of the gut mucosa and immune system. Therefore, these stressors can increase the susceptibility to diseases and reduce the growth performance, causing great economic losses (Osho and Adeola 2020; Y. Wang et al. 2023). In the past, antibiotic growth promoters were used to help to control immunosuppression; however, increasing concerns regarding antibiotic resistance have led to the application of alternative strategies (Williams, Verstegen, and Tamminga 2001; Osho and Adeola 2020).

Many natural feed additives, such as yeast, antioxidants and prebiotics, are being utilised to alleviate stress, improve gut health and enhance immune function in poultry (Q. Wang et al. 2021; Y. Wang et al. 2023). Yeast and its derivatives (live yeast, yeast extracts and yeast cell wall components) contain numerous bioactive compounds (Kim et al. 2022). Among them, β‐glucan, a yeast cell wall component, consists of the polymerisation of glucose through β‐1,3/1,6 glycosidic linkages and is widely studied in poultry, based on its beneficial effect on innate and acquired immunity (Shao et al. 2013; Anwar et al. 2017; Ezzat et al. 2024). In chickens, β‐glucan could mitigate inflammation by decreasing the levels of serum pro‐inflammatory cytokines, such as tumour necrosis factor‐α (TNF‐α), interleukin‐1β (IL‐1β) and interleukin‐6 (IL‐6) (Vetvicka and Oliveira 2014; Kim et al. 2022). In addition, β‐glucan increases the mRNA expression of anti‐inflammatory interleukin‐10 (IL‐10) in chickens (W. Wang et al. 2016). β‐Glucan could further improve the humoral immune response by elevating antibody responses against vaccines and higher levels of immunoglobulins in broiler chickens (Ezzat et al. 2024; Abd E Al‐Tawab, Elnaggar, and El‐Sissi). Furthermore, β‐glucan is noted to ameliorate the intestinal mucosal barrier impairment caused by *Salmonella typhimurium* challenge in broiler chickens by enhancing the mRNA expressions of tight junction (TJ) proteins, such as claudin‐1 and occludin (OCLN) (Shao et al. 2013).

Other potential bioactive compounds can be utilised in diets to improve immunity in poultry. Antioxidants are among the frequently administered compounds to maintain an effective antioxidant system and enhance immune response (Surai et al. 2019; Farahat et al. 2017). Antioxidants can help to mitigate oxidative stress by scavenging reactive oxygen species (ROS); therefore, they can maintain redox balance (Mishra and Jha 2019). By reducing oxidative stress, antioxidants play a crucial role in protecting immune cells and facilitating the proliferation and differentiation of B lymphocytes into antibody‐producing plasma cells (Farahat et al. 2017).

Characterised by their potent antioxidant properties, carotenoids are naturally occurring pigments in plants supporting various essential physiological functions, including enhancing immune response in animals (Nabi et al. 2020). The potential anti‐inflammatory effect of capsanthin (a major carotenoid of red pepper) was reported to reduce mRNA (IL‐6 and TNF‐α) and plasma levels (IL‐1β and IL‐6) of pro‐inflammatory cytokines during the LPS challenge in our previous study (Csernus et al. 2023). β‐Carotene was also reported to have a significant effect on male chickens’ immune status by improving the mRNA expression of *IgA* and jejunal mucosal *IgA*. In addition, it promoted the intestinal barrier function by increasing the mRNA expressions of intestinal barrier‐related proteins, such as zonula occludens‐1 (*ZO‐1*) and *ZO‐2* and *OCLN* (Hui et al. 2020).

Other bioactive compounds, anthocyanins are a group of flavonoids found in plants, such as plums, cherries and blackberries, that produce red, purple or blue colours (Changxing et al. 2018; Y. Wang et al. 2023). They have several pharmacological effects involving antioxidant, anti‐inflammatory and antibacterial activities (Castaneda‐Ovando et al. 2009; B. W. Lin et al. 2017). Anthocyanins could inhibit inflammation by suppressing the ileal contents of pro‐inflammatory cytokines (IL‐1β, IL‐6, IL‐8, TNF‐α, IFN‐β and IFN‐γ) during *Salmonella* challenge in chickens. In addition, anthocyanins mitigated intestinal barrier damage by upregulating the expression of TJ proteins, such as OCLN and ZO‐1, in *Salmonella*‐infected male broilers (Zhang et al. 2023).

Furthermore, several oligosaccharides (e.g., fructooligosaccharides, galactooligosaccharides or chitooligosaccharides) are often utilised as prebiotics in poultry nutrition (Y. Lin, Teng, and Olukosi 2022). These oligosaccharides are carbohydrates involving 3–10 sugar monomer units naturally occurring in plants (Mussatto and Mancilha 2007; Al‐Surrayai and Al‐Khalaifah 2022). Oligosaccharides are metabolised by the beneficial microorganisms in the colon; therefore, they can influence the gut microbiota, which is essential for nutrition, physiological processes, and immune function (Al‐Surrayai and Al‐Khalaifah 2022). Some of these compounds, for example, chitooligosaccharides, are reported to decrease the levels of pro‐inflammatory cytokines (IL‐1β and IFN‐γ) and increase the expression of OCLN, a TJ protein. Therefore, they could alleviate the immunological stress and intestinal barrier impairment induced by the lipopolysaccharide (LPS) challenge (Gu et al. 2022).

Bacterial LPS is a cell wall component of Gram‐negative bacteria and is often used as a model to study inflammatory responses in broiler chickens. LPS triggers the activation of the transcription factor nuclear factor kappa B (NF‐κB), which subsequently promotes the expression of pro‐inflammatory cytokines (Lee et al. 2017). As part of the innate and adaptive immune system, cytokines function as extracellular signals between cells during cellular immune responses (Kaiser and Stäheli 2008). IL‐1β and IL‐6 are key pro‐inflammatory cytokines (Dinarello 2000; Kambayashi et al. 1995). IL‐1β activates macrophages and T cells and has a role in inflammatory responses (Lotz et al. 1988; Klasing 1988), and the up‐regulation of IL‐6 can be interpreted as an acute‐phase reaction (Hong et al. 2006). LPS also affects gut permeability through the downregulation of TJ proteins (Lee et al. 2017). TJ proteins play a crucial role in the mechanical barrier of the intestinal epithelium, helping to confine harmful bacteria within the intestines and preventing the transfer of large molecules from the intestinal lumen to the epithelial cells (Ballard, Hunter, and Taylor 1995; Guo et al. 2023). TJ consists of multiprotein complexes containing cytosolic and transmembrane proteins. Zonula occludens‐1 (ZO‐1) has an important role in establishing and maintaining the epithelial barrier. It interacts with transmembrane proteins such as OCLN and claudins, as well as the actin cytoskeleton, thereby connecting these elements to properly organise the TJ complex (Fanning et al. 1998; Fanning et al. 2002). OCLN and the claudin family are key components in regulating the functionality of the intestinal epithelial barrier (Fanning et al. 1998).

Since gut health is closely associated with immune status and plays a vital role in improving animal production, this research aimed to investigate the effects of natural substances, such as β‐glucan, carotenoids, oligosaccharides and anthocyanins, on immune response and TJ proteins in broiler chickens under *Escherichia coli* LPS challenge. The bioactive compounds used in this study are derived from waste materials of the food industry, which suggests that they could be further utilised if their beneficial effects on intestinal health and immunity in broilers are confirmed. Depending on the source and structure, the physiological effects of these bioactive compounds can vary, making it essential to further investigate their impact (Guo et al. 2023). The positive effects of the bioactive substances used are expected to result in lower plasma levels of pro‐inflammatory cytokines (IL‐1β and IL‐6) and increased levels of immunoglobulin G (IgG) and ileal TJ proteins, including ZO‐1, OCLN 1 and OCLN 2, during the LPS challenge.

The comparative assessment of plasma levels of TJ proteins (ZO‐1, OCLN 1 and OCLN 2) was also conducted to elucidate the systemic effects of the natural bioactive agents on gut barrier integrity in broiler chickens, both with and without LPS challenge. Since the ileal measurements provide localised insights into intestinal barrier function under an inflammatory response, analysing plasma levels allows us to evaluate the potential systemic impact, with TJ proteins serving as important biomarkers.

## 2 Materials and Methods

### 2.1 Preparation of Extracts

β‐Glucan was originated from *Saccharomyces cerevisiae*. Carotenoids were extracted from Hungarian red sweet pepper powder. Extraction was carried out with HPLC as previously described (Nagy et al. 2017). Major carotenoid compounds were determined by Diode Array Detector detection at 460 and 350 nm. Based on the HPLC profile, carotenoid compounds with the greatest areas were the following: capsanthin, cis‐capsanthin, β‐carotene and zeaxanthin (Csernus et al. 2020). Oligosaccharides with high arabino‐galactose content were extracted from Hungarian red sweet pepper retained from industrial food waste. HP 5890 gas chromatograph (Hewlett‐Packard, Agilent Technologies Inc., Palo Alto, CA, USA) with SP 2380 capillary column (30 m, 0.25 mm, 0.2 m) (Merck Life Science KGaA, Darmstadt, Germany) and flame ionisation detector were applied for detection of the monomer units of oligosaccharides as glucose, arabinose, xylose, galactose and mannose were identified (Csernus et al. 2020). Anthocyanins were extracted from Hungarian sour cherry using a VWR‐Hitachi ChromasterUltraRs UHPLC (Hitachi, Tokyo, Japan) with a Phenomenex Kinetex column (2.6 m, XB‐C18, 100 Å, 100 4.6 mm) (Phenomenex, Torrance, CA, USA) as published earlier (Nemes et al. 2018). The main anthocyanin compounds were cyanidin‐3‐O‐glucosyl‐rutinoside, cyanidin‐3‐O‐rutinoside and cyanidin‐3‐O‐monoglucoside, which were determined according to Homoki et al. (2016).

### 2.2 Birds, Housing and Experimental Diets

A total of 900 one‐day‐old mixed‐sex broiler chickens (Ross 308) were used in a 27‐day feeding trial. Chickens were allocated to five treatments, each in three‐floor pens (replicates), with 60 broilers per pen covered with wood shavings in a thermostatically controlled house at a stocking density of 650 cm^2^/bird. Males and females were equally distributed in each pen. Diets were based on corn, wheat and soybean meal. The five treatments used in this experiment were a basal diet (control group) (Table 1), a basal diet supplemented with 0.05% β‐glucan, and a basal diet supplemented with 0.5% carotenoids, oligosaccharides or anthocyanins. The inclusion of feed supplements was applied based on studies demonstrating that these concentrations effectively enhance immune function, reduce inflammatory responses, and improve gut health in poultry (Shang et al. 2015; W. Wang et al. 2016). The basal diets (pre‐starter, starter and grower) were fed in mashed form. Feed and clean drinking water were available ad libitum throughout the entire feeding trial. The chickens were exposed to light according to Olanrewaju et al. (2006) as follows: 23L:1D during the first 7 days, 20L:4D between 8 and 27 days (L = light, D = dark).

**TABLE 1:** Composition, nutrient and energy content of the basal diet.

	Value		
Composition, %	Pre‐starter (Days 1–9)	Starter (Days 10–21)	Grower (Days 22–27)
Corn	33	34	33
Wheat	27	29	31
Soybean meal, solvent extracted (46.0% CP)	29	24	20
Soybean meal, extruded (46.0% CP)	4	6	4
Sunflower meal, extracted		1	3
Feed yeast	1		
DDGS		1	3
Plant fats	2	1	3
Premix	4	4	3
Total	100	100	100
Energy and nutrient content			
Dry matter, %	89.06	89.03	89.15
AME_n_ poultry, MJ/kg	12.23	12.47	12.81
Crude protein, %	21.58	20.28	19.05
Crude fat, %	4.61	4.83	6.22
Crude fibre, %	3.37	3.51	3.7
Lysine, %	1.37	1.27	1.17
Methionine, %	0.57	0.54	0.53
Methionine + cysteine, %	0.94	0.9	0.87
Calcium, %	0.85	0.73	0.71
Phosphorus, %	0.63	0.55	0.52

### 2.3 Sample Collection

The experiment was conducted using male broiler chickens exclusively to minimise variability related to sex differences in immune response and TJ proteins, thereby allowing for a more accurate assessment of the efficacy of feed supplements during the LPS challenge. This practice is also consistent with other studies in the field, which also focus on male broiler chickens (Shang et al. 2015; Barekatain et al. 2019). Male chickens were randomly selected for sample collection. On Day 25 of the experiment, blood samples (*n* = 6 per treatment) were collected into EDTA‐coated vacutainer tubes from the brachial vein of broiler chickens. Plasma was separated by centrifugation (4000 RPM × 10 min). On Day 26, a critical period of immune development, six broilers from each treatment were injected with 2 mg/kg live weight *E. coli* O55:B5 LPS (L2880, Sigma, St. Louis, MO, USA), intraperitoneally. In the control group, another six chickens were inoculated with 2 mL/kg live weight isotonic saline solution (B. Braun, Budapest, Hungary) in the same way (Shang et al. 2015). This timing of the LPS challenge aligns with existing literature, which frequently focuses on similar age points such as Days 25 and 27, indicating that this developmental stage is particularly relevant for evaluating the impact of feed supplements on immune modulation (W. Wang et al. 2016; M.‐Y. Wang et al. 2022). Twelve hours after the LPS injection (on Experimental Day 27), all of the injected birds were sacrificed by cervical dislocation (M. Alizadeh, Rodriguez‐Lecompte, et al. 2016). Blood and terminal ileum tissues were immediately collected from each chicken. The whole blood was centrifuged, then plasma and ileal samples were snap‐frozen in liquid nitrogen and stored at −80°C for further measurements.

### 2.4 Measurement of Plasma Cytokine and IgG Concentrations

Plasma IL‐1β, IL‐6 cytokines and IgG were measured according to the manufacturer's protocol. Blanks, standards and samples were analysed in duplicate. An HTX Synergy Multi‐Mode Microplate Reader (Bio‐Tek Instruments, Inc., Winooski, VT, USA) was applied to measure the absorbance at 450 nm and a linear standard curve fit was developed using Gen5 Microplate Data Analysis Software (Bio‐Tek Instruments, Inc., Winooski, VT, USA). Coefficients of variation for intra‐assay were 6.78%, 4.62% and 7.28% for IL‐1β, IL‐6 and IgG, respectively.

### 2.5 Determination of Ileal and Plasma ZO‐1, OCLN 1 and OCLN 2 Levels

Proteins related to the TJ functions were determined in plasma and the terminal part of the ileum tissue. Baseline levels of TJ proteins were also analysed from plasma samples collected on Day 25, before the LPS challenge. Ileum (25 mg) was homogenised in 250 µL of 10 mM PBS solution (pH 7.4) using a tissue homogeniser (VWR International Kft, Hungary). ZO‐1, OCLN 1 and OCLN 2 were measured using a commercially available Chicken ELISA Kit (Sunlong Biotech, Ltd., Hangzhou, Zhejiang, China). Blanks, standards and samples were analysed in duplicate. A BMG Labtech SPECTROstar Nano Microplate Reader (BMG Labtech, Ortenberg, Germany) was used to determine absorbance at 450 nm, and a four‐parameter logistic curve fit was developed using SPECTROstar Nano MARS Software (BMG Labtech, Ortenberg, Germany). Coefficients of variation for intra‐assay were 3.15%, 8.85% and 6.79% for ileal ZO‐1, OCLN 1 and OCLN 2, respectively. Coefficients of variation for intra‐assay were 8.18%, 7.3% and 10% for plasma ZO‐1, OCLN 1 and OCLN 2, respectively. Coefficients of variation for inter‐assay were 11%, 7.25% and 11% for plasma ZO‐1, OCLN 1 and OCLN 2, respectively.

### 2.6 Statistical Analysis

The statistical analysis was carried out using the GraphPad Prism 8.0.1 software. One‐way analysis of variance (ANOVA) was applied. The normality of data was tested by the Shapiro–Wilk test. The equality of variance was checked by Bartlett's test. The main effects of the natural substances on immune response and TJ proteins were analysed using a one‐way analysis of variance, and Tukey's test was used to compare the means of each treatment. In the case of a lognormal distribution of data, Dunn's test, in the case of unequal variances, Tamhane's T2 test was applied. Differences among treatments were considered significant at *p* < 0.05.

## 3 Results

### 3.1 Plasma Cytokine Levels

**FIGURE 1:**
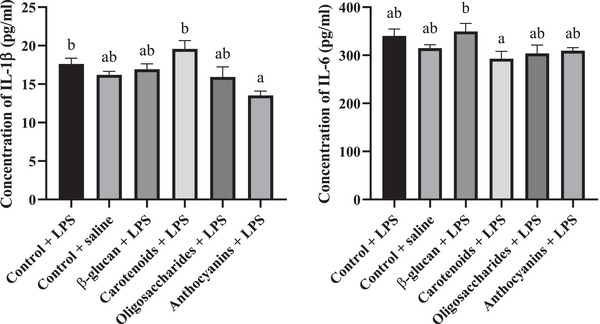
(A) Plasma interleukin‐1β (IL‐1β) and (B) interleukin‐6 (IL‐6) levels of broiler chicken fed control diet under LPS challenge, control diet under isotonic saline challenge, a diet supplemented with 0.05% β‐glucan, a diet supplemented with 0.5% carotenoid, a diet supplemented with 0.5% oligosaccharide and a diet supplemented with 0.5% anthocyanin under LPS challenge (*n* = 6/treatment). Error bars represent means + standard errors of the mean. Groups that do not share a letter are significantly different (*p* < 0.05).

Plasma IL‐1β and IL‐6 levels are shown in Figure 1. The concentrations of IL‐1β were 17.6 pg/mL in the LPS‐treated control group, 16.2 pg/mL in saline‐treated control birds, 16.9 pg/mL in the β‐glucan‐treated group, 19.6 pg/mL in carotenoid‐treated chickens, 16 pg/mL in oligosaccharide‐treated birds and 13.5 pg/mL in anthocyanin‐treated broilers. The results showed that anthocyanins decreased plasma IL‐1β level (*p* = 0.047) compared to the LPS‐injected control birds. Anthocyanin treatment also showed a lower level of IL‐1β compared to the carotenoid treatment as well (*p* = 0.0414). The levels of IL‐6 were 340.3 pg/mL in the control LPS‐injected birds, 314.8 pg/mL in control saline‐injected birds, 349.5 pg/mL in β‐glucan treatment, 292.7 pg/mL in carotenoid treatment, 303.8 pg/mL in oligosaccharide treatment and 309.3 pg/mL in anthocyanin treatment. The applied supplementations did not affect the level of IL‐6 compared to the LPS‐injected control group, but carotenoids showed lower expression of the cytokine compared to the β‐glucan treatment (*p* = 0.0466).

### 3.2 Plasma IgG Concentration

**FIGURE 2:**
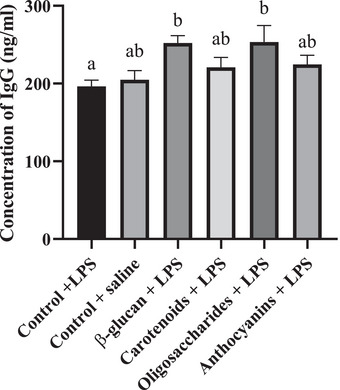
Plasma immunoglobulin G (IgG) level of broiler chicken fed control diet under LPS challenge, control diet under isotonic saline challenge, a diet supplemented with 0.05% β‐glucan, a diet supplemented with 0.5% carotenoid, a diet supplemented with 0.5% oligosaccharide and a diet supplemented with 0.5% anthocyanin under LPS challenge (*n* = 6/treatment). Error bars represent means + standard errors of the mean. Groups that do not share a letter are significantly different (*p* < 0.05).

Concentrations of chicken plasma IgG can be seen in Figure 2. Levels of IgG were the following: 196.4 ng/mL in the control (LPS) group, 205 ng/mL in the control (saline) group, 252.1 ng/mL in the β‐glucan group, 220.8 ng/mL in the carotenoid group, 253.5 ng/mL in the oligosaccharide group and 224.6 ng/mL in the anthocyanin group. β‐glucan (*p* = 0.0382) and oligosaccharides (*p* = 0.0449) increased the plasma IgG levels compared to the control group under the *E. coli* LPS challenge.

### 3.3 Ileal ZO‐1, OCLN 1 and OCLN 2 Levels

**FIGURE 3:**
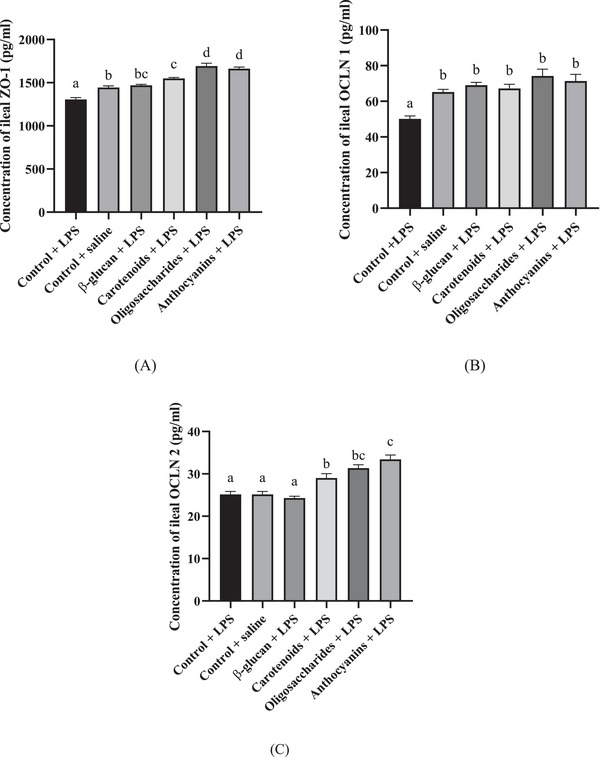
(A) Ileal zonula occludens‐1 (ZO‐1), (B) occludin 1 (OCLN 1) and (C) occludin 2 (OCLN 2) levels of broiler chicken fed control diet under LPS challenge, control diet under isotonic saline challenge, a diet supplemented with 0.05% β‐glucan, a diet supplemented with 0.5% carotenoid, a diet supplemented with 0.5% oligosaccharide and a diet supplemented with 0.5% anthocyanin under LPS challenge (*n* = 6/treatment). Error bars represent means + standard errors of the mean. Groups that do not share a letter are significantly different (*p* < 0.05).

Ileal ZO‐1, OCLN 1 and OCLN 2 levels are shown in Figure 3. The levels of ZO‐1 were 1305.5 pg/mL in the control (LPS) group, 1443.6 pg/mL in the control (saline) group, 1469.9 pg/mL in the β‐glucan group, 1548.5 pg/mL in the carotenoid group, 1692.4 pg/mL in the oligosaccharide group and 1662.3 pg/mL in the anthocyanin group. All of the applied supplementations increased the level of ZO‐1 compared to the control (LPS) group (*p* < 0.0001).

Ileal OCLN 1 concentrations were 50.13 pg/mL in the control (LPS) group, 65.2 pg/mL in the control (saline), 69 pg/mL in the β‐glucan supplementation, 67.2 pg/mL in the carotenoid supplementation, 74.2 pg/mL in the oligosaccharide supplementation and 71.4 pg/mL in the anthocyanin supplementation. All treatments (β‐glucan: *p* = 0.0002; carotenoids: *p* = 0.0009; oligosaccharides and anthocyanins: *p* < 0.0001) increased the concentration of ileal OCLN 1 compared to LPS‐injected control birds.

The concentrations of ileal OCLN 2 were 25.1 pg/mL in the control (LPS) treatment, 25.1 pg/mL in the control (saline) treatment, 24.3 in the β‐glucan treatment, 29 pg/mL in the carotenoid treatment, 31.3 pg/mL in the oligosaccharide treatment and 33.4 pg/mL in the anthocyanin treatment. The results showed that carotenoids (*p* = 0.0233), oligosaccharides (*p* = 0.0001) and anthocyanin (*p* < 0.0001) supplementations increased the levels of ileal OCLN 2 compared to the control birds under LPS challenge.

### 3.4 Plasma ZO‐1, OCLN 1 and OCLN 2 Levels

**FIGURE 4:**
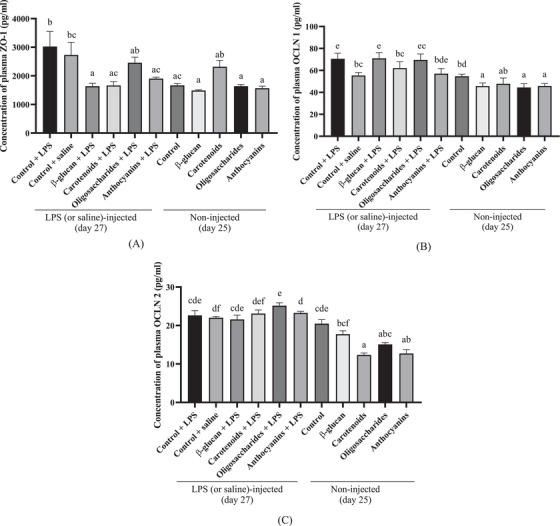
(A) Plasma zonula occludens‐1 (ZO‐1), (B) occludin 1 (OCLN 1) and (C) occludin 2 (OCLN 2) levels of broiler chicken fed control diet under LPS challenge, control diet under isotonic saline challenge, a diet supplemented with 0.05% β‐glucan, a diet supplemented with 0.5% carotenoid, a diet supplemented with 0.5% oligosaccharide and a diet supplemented with 0.5% anthocyanin under LPS‐challenge at experimental Day 27 (*n* = 6/treatment). TJ proteins are shown in the applied treatments before LPS‐challenge at experimental Day 25 (*n* = 6/treatment) compared to plasma TJ levels under LPS challenge at experimental Day 27. Error bars represent means + standard errors of the mean. Groups that do not share a letter are significantly different (*p* < 0.05).

Plasma ZO‐1, OCLN 1 and OCLN 2 concentrations measured in LPS (or saline)‐injected treatments on Day 27 and non‐injected treatments (on Day 25) are shown in Figure 4. All groups were compared to each LPS‐injected and non‐injected group as well.

In LPS‐challenged groups, levels of plasma ZO‐1 were 3026.1 pg/mL in the control (LPS) group, 2727.8 pg/mL in the control (saline) group, 1638.8 pg/mL in the β‐glucan‐treated group, 1664.8 pg/mL in the carotenoid‐treated group, 2457.4 pg/mL in the oligosaccharide‐treated group and 1905.67 pg/mL in the anthocyanin‐treated group. Concentrations of plasma ZO‐1 were lower in the non‐injected control (*p* = 0.0038), β‐glucan (*p* = 0.0006), oligosaccharides (*p* = 0.0029) and anthocyanin (*p* = 0.0014) treatments compared to LPS‐injected control birds.

In LPS‐injected birds, the concentration of plasma OCLN 1 was 70.5 pg/mL in the control (LPS) group, 55.4 pg/mL in the control (saline) group, 70.9 pg/mL in the β‐glucan‐fed birds, 62.2 pg/mL in the carotenoid‐fed birds, 69.6 pg/mL in the oligosaccharide‐fed birds and 57 pg/mL in the anthocyanin‐fed birds. The LPS‐injected carotenoid (*p* = 0.0454) and anthocyanin (*p* < 0.0001) treatments resulted in decreased levels of OCLN 1 compared to the LPS‐injected control birds. All of the non‐injected groups (*p* < 0.0001) showed decreased concentrations of OCLN1 compared to LPS‐injected control birds. The LPS‐injected β‐glucan (*p* < 0.0001), oligosaccharides (*p* < 0.0001) and anthocyanins (*p* = 0.0013) showed higher concentrations of OCLN 1 compared to their non‐injected pairs (at Day 25).

Under LPS challenge, concentrations of plasma OCLN 2 were 22.6 pg/mL in the control (LPS) group, 22 pg/mL in the control (saline) group, 21.6 pg/mL in the β‐glucan‐treated group, 23.1 pg/mL in the carotenoid‐treated group, 25.2 pg/mL in the oligosaccharide‐treated group and 23.3 pg/mL in the anthocyanin‐treated group. The LPS‐injected and supplemented groups had no significant effects on plasma OCLN 2 levels compared to the (LPS) control group. Among the non‐injected groups (on Day 25), carotenoids (*p* = 0.0084) and anthocyanins (*p* = 0.0069) showed lower concentrations of OCLN 2 compared to the LPS‐injected control birds (on Day 27). The LPS‐injected carotenoid‐ (*p* = 0.0005), oligosaccharide‐ (*p* < 0.0001) and anthocyanin‐treated (*p* = 0.0017) birds (Day 27) showed increased concentrations of OCLN 2 compared to their non‐injected groups (on Day 25).

## 4 Discussion

In this study, the impacts of selected natural substances, such as β‐glucan, carotenoids, oligosaccharides and anthocyanins, on immune response and gut barrier integrity were examined in chickens under *E. coli* LPS challenge.

The immune system defends the host from external pathogens through the process of recognition and response. During immune responses, including infection, injury or inflammation, cytokines are the main regulators (Kyoung et al. 2023). Bacterial LPS induces the expression of pro‐inflammatory cytokines. However, plasma levels of IL‐1β and IL‐6 did not change in the LPS‐challenged control group compared to the saline‐injected control birds in our study. In contrast, LPS immunisation increased the splenic and ileal IL‐1β expressions at the mRNA level in our previous research; therefore, the *E. coli* LPS inoculation could induce an acute‐phase response (Csernus et al. 2020). Although plasma proteins originate from the blood, they accumulate in the impacted tissues. Hence, understanding the complex interplay of vascular alterations, molecular signalling pathways and local cellular responses is essential for effectively managing and eradicating infections (French et al. 2020). On the other hand, the discrepancy between mRNA and protein expression can be attributed to post‐transcriptional processes, including mRNA stability and the extended half‐life of the protein, which can be influenced by post‐translational modifications that frequently affect protein levels (Ideker et al. 2001).

Among the examined bioactive compounds, the supplementation of anthocyanins could decrease the level of pro‐inflammatory IL‐1β compared to the LPS‐challenged control group. In our previous study, anthocyanins were also shown to reduce the splenic IL‐1β at the gene expression level (Csernus et al. 2020). Anthocyanin‐rich fragments also resulted in lower mRNA levels of IL‐1β in mice (L. Li et al. 2014). Similar to our results, Wu, Wang, and Qi (2016) reported decreased plasma levels of IL‐1β in broilers at 20 days of age when dietary supplementation was applied. The authors suggested that dietary supplementation could modify the secretion of IL‐1β and modulate the functions of macrophages. Dietary bilberry anthocyanins could further reduce jejunal and plasma contents of IL‐1β in chickens during *S. typhimurium* infection, suggesting their beneficial role in alleviating inflammation (Zhang et al. 2023). These results may be attributed to the anti‐inflammatory and antioxidant properties of anthocyanins, which potentially inhibit the pro‐inflammatory signalling pathways and mitigate oxidative stress (Carvalho et al. 2015). These findings suggest that anthocyanins could serve as a dietary intervention to enhance immune response in broiler chickens.

In the current study, none of the applied supplementations changed the level of pro‐inflammatory IL‐6 compared to LPS‐challenged control birds. However, carotenoids lowered the level of IL‐6 compared to the β‐glucan treatment. Similarly, xanthophyll (a class of carotenoids) supplementation at 20 and 40 mg/kg in feed decreased the mRNA level of hepatic IL‐6 in hens at Experimental Day 35 (Gao et al. 2012). In our previous study, carotenoids reduced the mRNA level of splenic IL‐6 in broiler chickens under the *E. coli* LPS‐challenge (Csernus et al. 2020). Capsanthin, another carotenoid compound, has also inhibited IL‐6 expressions at both mRNA and protein levels in male broilers under LPS immunisation, indicating its potential anti‐inflammatory impact (Csernus et al. 2023).

Natural antibodies are part of humoral immunity, and among them, IgG is a predominant form in birds during the secondary antibody response (Davison, Magor, and Kaspers 2008). Among the applied feed additives, β‐glucan and oligosaccharide supplementations increased the level of plasma IgG in our study. In agreement with this, Bakhshalinejad, Moghaddam Kakhki, and Zoidis (2018) reported an increased serum IgG level of chickens under sheep red blood cell challenge when a dietary treatment was applied. Another study also reported increased serum IgG levels when a feed supplementation was used (Cai et al. 2012). The authors discussed an improved humoral immune response of chickens in response to feed supplementation. X. H. Li et al. (2016) reported that adding yeast cell wall powder at 1 g/kg to the feed increased ileal IgG in 21‐day‐old chickens, thus yeast cell wall promoted the ileal immune status. β‐Glucans, primarily found in the cell walls of yeast, stimulate immune cells such as macrophages and lymphocytes, promoting the production of antibodies, including IgG (Schwartz and Vetvicka 2021). Similar to our results, the plasma IgG level of 21‐day‐old chickens was also higher when feed was supplemented by xylooligosaccharides at 2 mg/kg, and the authors suggested that xylooligosaccharides could improve immune functions (Yuan et al. 2018). Ileal IgG contents in broilers were also increased at 21 and 42  days of age, and jejunal IgG content was also higher at 21 days of age when mannan‐oligosaccharides were added to the feed at the levels of 0.5, 1 and 1.5 g/kg. The authors noted that mannan‐oligosaccharides could enhance the intestinal immunity by affecting jejunal and ileal intestinal cells to secrete other immunoglobulins (Zhou et al. 2019).

In this study, the effects of natural substances on ileal TJ proteins, such as ZO‐1, OCLN 1 and OCLN 2 in broiler chickens during LPS‐challenge were also examined. TJ proteins are crucial components of the intestinal epithelial mechanical barrier (Guo et al. 2023). The epithelial barrier is important for maintaining intestinal homeostasis, as it facilitates the absorption of water and essential nutrients while serving as a protective shield against potential harmful stressors, including bacteria, viruses and undesirable substances that may enter the intestinal tract (A. Alizadeh, Akbari, et al. 2016; Stevens et al. 2021). In a previous study, *E. coli* LPS challenge decreased the mRNA level of ileal ZO‐1 in broiler chickens and resulted in impaired intestinal barrier function (Chen et al. 2018). As we predicted, LPS also downregulated the ileal ZO‐1 and OCLN 1 compared to the saline‐injected control group in this study, which suggests that LPS could disrupt the intestinal barrier of chickens (Gu et al. 2022).

Among the applied bioactive substances, β‐glucan increased the levels of ZO‐1 and OCLN 1 compared to the LPS‐injected control birds. Similarly, dietary yeast cell wall increased the gene expression levels of TJ proteins, such as claudin‐1 and claudin‐2 (*CLDN1* and *CLDN2*), TJ protein‐1 (*TJP*) and mucin‐1 (*MUC1*) and suggested that dietary yeast cell wall could modulate the intestinal integrity and support the intestinal health (Kyoung et al. 2023). In another study, β‐1,3/1,6‐glucan also increased the mRNA levels of TJ proteins in the jejunum, including TJ proteins, such as *OCLN* and *CLDN1*. Therefore, the authors suggested that β‐glucan may be involved in mitigating the enhanced gut permeability induced by the *Salmonella* challenge (Shao et al. 2013).

In our study, carotenoids further increased the levels of all the examined TJ proteins (ZO‐1, OCLN 1 and OCLN) in the ileum of broiler chickens under LPS challenge. Previous study noted that lutein supplementations resulted in higher mRNA expressions of jejunal *OCLN*, *CLDN* and *ZO‐1* and suggested that the addition of lutein could reverse the downregulation of TJ proteins (M.‐Y. Wang et al. 2022). Hence, carotenoids can be involved in controlling the barrier function by regulating the TJ. Carotenoids are recognised for their antioxidant and anti‐inflammatory properties, as well as their ability to modulate microbiota composition. Consequently, these effects contribute to the improvement of barrier dysfunction (Cheng et al. 2021).

In this study, oligosaccharides further increased the levels of all the studied ileal TJ proteins. Previous studies reported similar results that chitooligosaccharide supplementation enhanced the mRNA levels of jejunal *OCLN* and *CLDN2*, in LPS‐challenged hens (Gu et al. 2022). Similarly, xylooligosaccharides also upregulated the jejunal *ZO‐1* and *OCLN* mRNA expressions in broilers, indicating their alleviating effects on gut barrier impairment (Q. Wang et al. 2021). Furthermore, anthocyanins improved the intestinal barrier function by elevating levels of ileal ZO‐1, OCLN 1 and OCLN 2. Similarly, anthocyanin supplements also induced the relative mRNA expression of ZO‐1 in broilers. Therefore, anthocyanins mitigated the LPS‐induced intestinal damage due to their antioxidant effect (Y. Wang et al. 2023).

In this study, the potential systemic effects of bioactive compounds were also investigated, and plasma concentrations of TJ proteins were measured. TJ‐associated proteins can be used as blood biomarkers since they are transferred to the circulation after disruption (Santos et al. 2021). In most instances, plasma levels of TJ proteins were notably higher in LPS‐challenged control broiler chickens compared to the non‐challenged treatment groups in our study. Furthermore, plasma levels of OCLN 1 were higher in LPS‐injected β‐glucan, oligosaccharides and anthocyanins‐treated groups compared to their non‐injected treatment pairs. Plasma concentrations of OCLN 2 were higher in the LPS‐injected carotenoids, oligosaccharides and anthocyanin treated birds compared to their non‐injected group pairs. This increase may suggest a disruption in gut barrier integrity in response to LPS exposure, indicating an inflammatory response that may compromise the functionality of the intestinal barrier.

## 5 Conclusions

In conclusion, the selected natural bioactive compounds, such as β‐glucan, carotenoids, oligosaccharides and anthocyanins, can be effective in mitigating inflammation and impaired gut barrier function in broiler chickens. Anthocyanins and carotenoids are suggested to be used for alleviating inflammation by decreasing pro‐inflammatory cytokines. β‐Glucan and oligosaccharides can be useful to enhance humoral immune response. All bioactive agents can be applied for improving the intestinal health, as all compounds could improve intestinal barrier impairment in broiler chickens induced by the LPS challenge.

## Author Contributions

**Brigitta Csernus**: data curation, investigation, methodology, software, writing–original draft. **Georgina Pesti‐Asbóth**: data curation, investigation, methodology, software, writing–original draft. **Judit Remenyik**: data curation, funding acquisition, investigation, supervision. **Sándor Biró**: conceptualization, formal analysis, funding acquisition, project administration, resources. **László Babinszky**: conceptualization, formal analysis, funding acquisition, writing–review and editing. **László Stündl**: conceptualization, funding acquisition, project administration, supervision. **János Oláh**: investigation, resources, supervision. **Nóra Vass**: investigation, methodology, writing–review and editing. **Levente Czeglédi**: conceptualization, data curation, formal analysis, supervision, validation, writing–review and editing.

## Ethics Statement

The experiments were confirmed by the University of Debrecen Committee of Animal Welfare, Hungary (Permit number: DEMAB/12‐7/2015). The authors confirm that the ethical policies of the journal, as noted on the journal's author guidelines page, have been adhered to and the appropriate ethical review committee approval has been received. The authors confirm that they have followed EU standards for the protection of animals used for scientific purposes and feed legislation.

## Conflicts of Interest

The authors declare no conflicts of interest.

### Peer Review

The peer review history for this article is available at https://publons.com/publon/10.1002/vms3.70175.

## Data Availability

The original data of the paper are available upon request from the corresponding author.
